# Treatment of Severe Steroid-Refractory Acute-Graft-vs.-Host Disease With Mesenchymal Stem Cells–Single Center Experience

**DOI:** 10.3389/fbioe.2018.00093

**Published:** 2018-07-24

**Authors:** Maja Česen Mazič, Lenart Girandon, Miomir Kneževič, Simona L. Avčin, Janez Jazbec

**Affiliations:** ^1^Department for Pediatric Hematology and Oncology, University Children Hospital Ljubljana, Ljubljana, Slovenia; ^2^Educell Ltd., Ljubljana, Slovenia

**Keywords:** immunosuppressive therapy, stem cell, pediatrics, GvHD, regeneration, remission induction

## Abstract

The most effective treatment of steroid refractory acute graft vs. host disease (aGvHD) is not yet established and mesenchymal stem cells (MSC) appear to be a promising therapy for the condition. We report single center case series of three patients, who underwent allogeneic hematopoietic cell transplantation and later developed steroid refractory graft-vs.-host disease, treated with MSC infusions. Two patients achieved complete remission and one patient partial remission of skin and/or gastrointestinal aGvHD. We demonstrated application of MSC for treatment of severe steroid refractory aGvHD is feasible in clinical practice. Detailed description of patient's features and MSC production protocol is crucial for future comparison on efficacy and safety of cell-based therapies. However, for any substantial conclusions regarding efficacy of MSC higher patient numbers will be required.

## Introduction

Graft vs. Host Disease (GvHD) is a significant cause of mortality and morbidity after allogeneic hematopoietic stem cell transplantation, with incidence ranging from 10 to 80%. First line therapy are high dose systemic corticosteroids, with durable complete response is 30 to 40% of patients, while others develop steroid-refractory acute GvHD (Deeg, 2007; Munneke et al., 2016). Steroid refractory aGvHD has a high mortality rate (up to 80%) despite intensified treatment with additional immunosuppressive agents, often with poor response rate and increased risk for toxic and infectious complications due to profound immunosuppression and long lasting GvHD. Surviving patients often develop chronic GvHD, which reduces life expectancy, performance and quality of life. There is presently no consensus as to the salvage treatment in acute steroid-refractory GvHD (Munneke et al., 2016).

Le Blanc et al. described in 2004 first use of mesenchymal stem cells (MSC) for treatment of 9-year-old boy with steroid refractory grade IV gut and liver aGvHD after HLA-matched unrelated HSCT and reported his complete and repeated recovery after MSC reinfusion (Le Blanc et al., 2004). Many safety and efficacy studies and case series published afterwards (Ringden et al., 2006; Le Blanc et al., 2008; Muller et al., 2008; von Bonin et al., 2009; Lucchini et al., 2010; Ball et al., 2013; Kuçi et al., 2016) confirmed patients with steroid refractory aGvHD can benefit from MSC infusions without adverse effect. In 2014 we developed a protocol for production and application of MSC from bone marrow of a third-party donor, encouraged by their efficacy and lack of availability through participation in a study or compassionate use program for MSC.

MSC are multipotent cells present in many different tissues (bone marrow, placenta, umbilical cord, dental pulp, adipose tissue) and capable of differentiation into several different cell types (chondrocytes, adipocytes, osteoblast, and myocytes). They harbor significant self-renewal potential (Dominici et al., 2006). Minimal definition criteria for MSCs include the capacity to adhere to plastic, the expression of CD105, CD73, and CD90, lack of CD45, CD34, CD14, HLA-DR, CD11b, CD79a, or CD19 expression, and the ability to differentiate into osteoblasts, adipocytes and chrondroblasts *in vitro* (Dominici et al., 2006).

MSC are involved in process of tissue damage repair and regulation of inflammation, functions important for treatment of aGvHD. Immunomodulation exerted by MSCs is mediated by secretion of different growth factors and expression adhesion molecules for cell-to-cell interactions (Ma et al., 2014). Source of MSCs and manufacturing protocol can have a significant effect on expression of MSC surface antigens and paracrine factor secretion and change final capacity of MSC to ensue desired effect (Bianco et al., 2008; Karp and Leng Teo, 2009). For this reason reporting detailed description of production and clinical protocols is crucial for understanding effect of MSC in patients with steroid refractory aGvHD.

Final effect of MSC depends also on specific pathological conditions of the recipient, thus identification of patients that respond to MSC will be important field of future research. Dazzi et al. showed that MSCs should undergo apoptosis by recipient cytotoxic cells, process essential to initiate MSC-induced immunosuppression by release of indoleamine 2,3-dioxygenase from activated phagocytes (Galleu et al., 2017). IDO mediated immunosuppression, through apoptosis of effector cells or up regulation of regulatory T cells, is important effect of MSC after they have been licensed by IFN-γ released by activated T cells in aGvHD (Dunavin et al., 2017).

## Patients and methods

### Patients

Three consecutive pediatric patients with grade IV steroid refractory aGvHD were treated with MSC between September 2014 and January 2017 in single (only) pediatric HSC transplant center in Slovenia.

First patient was diagnosed at the age of 11 years with acquired severe aplastic anemia that was treated with immunotherapy. In the course of the disease significant paroxysmal nocturnal hemoglobinuria clone evolved. He was managed symptomatically with red blood cell transfusions for 2 years, since hemolysis was leading symptom. His condition was complicated with thrombosis of intracranial venous sinus. At that time therapy with Eculizumab was introduced. He was transfusion free and without thrombotic events on Eculizumab. Bone marrow transplantation from matched unrelated donor was performed 2 years later (October 2013), at the age of 16 years, as an attempt to achieve permanent cure. From October 2013 until April 2014 three consecutive HSCT were done, due to repeated graft failure. Reduced intensity conditioning with Thiotepa, Fudarabine, Alemtuzumab; Alemtuzumab alone and Thiothepa, Cyclophosphamide and Thymoglobulin were performed, respectively. Hematopoetic stem cell (HSC) sources were matched unrelated donor (MUD) bone marrow, MUD double cord and MUD peripheral stem cell (apheresis) consecutively. Post-transplant prophylaxis with cyclosporine was changed to MMF due to side effects (extrapyramidal signs with dystonia and tremor). Engraftment occurred at day 35 after third transplantation and by day 65 patient developed generalized erythroderma, profuse vomiting and diarrhea (>10,000 ml/day); grade III aGvHD confirmed with skin and gastrointestinal (GIT) biopsy. Liver function tests remained within reference values. There was no response to high doses of systemic and local corticosteroids (Methylprednisolon 2 mg/kg iv, Budesonid) in combination with MMF and Tacrolimus. Targeted therapy with Infliximab and Alemtuzumab yield no results. Extracoporal photopheresis, Sirolimus, Imatinib and Methotrexate were also introduced without clinical effect.

By day 173 patient was cachectic, with grade III skin and gut aGvHD, distinctive ocular, nail and mouth lesions signaling progression to cGvHD. He was treated for repeated bacterial, viral, and invasive fungal infections.

After ethics committee approval and informed consent, MSC were isolated and multiplied from patient's father's bone marrow and infused at dose 2 × 10^6^/kg, on day 173, day 201, and day 271. Detailed procedure is described in methods. After first infusion marked improvement of skin and graft function was noticed, in terms of leucocyte and platelets counts. After second infusion, significant reduction of stool volume from >10,000 ml/d to 1,500 ml/day and improved endoscopic appearance of GIT mucosa, was noticed. Patient died 5 months later due to fulminant sepsis with uncontrolled pulmonary aspegillosis, CMV and adenovirus reactivation present before MSC infusion.

Second patient was diagnosed with high risk AML with CNS and testicular involvement at the age of 6 months. Since no HLA-matched unrelated donor was available, he was treated with intensive chemotherapy. He relapsed in bone marrow during maintenance therapy. After two courses of high dose chemotherapy, allogeneic HSC transplantation with minor HLA-mismatched unrelated donor was performed in second remission. Conditioning regimen consisted of Busulphan, Cyclophosphamide and Melphalan; Cyclosporin and Antithymocyte globulin was used for GVHD prophylaxis. Progressive respiratory failure manifested during engraftment period on day +25 with the need of intensive care treatment, mechanical ventilation and cardiovascular support. After exclusion of active respiratory infection and hemorrhage, idiopathic pneumonia syndrome (IPS) was considered the most probable etiology. Despite treatment with high dose systemic corticosteroids (Dexamethasone), respiratory failure progressed. With imminent need for extracorporeal membrane oxygenation, Etanercept was used due to high levels of circulating tumor necrosis factor alpha. After resolution of acute respiratory event, pulmonary aspergillosis was diagnosed (day +70) and treated with Voriconazole; low dose Dexamethasone was tapered, Cyclosporin continued. By day +238 patient developed generalized erythroderma, profuse vomiting and diarrhea (>30 ml/kg/day); grade III skin and GIT aGvHD was confirmed with biopsy of the involved organs. Systemic and local corticosteroids (Methylprednisolon 2 mg/kg iv, Budesonid) were introduced as first line therapy, while patient was still receiving Cyclosporin. Because of poor response to corticosteroids, progressive pulmonary aspergillosis and adenoviral reactivation, bone marrow derived MSC from unrelated third party donor, was applied as second line therapy for steroid resistant GvHD in dose of 2 × 10^6^ MSC/kg on day +280 and +287. After second application patient passed normal stool, skin was without pathological changes. On day +294 he was admitted in the ICU because of pulmonary aspergillosis progression with subsequent central nervous system involvement, patient died 2 months later without clinical signs of aGvHD.

Third patient was diagnosed with standard risk ALL at the age of 6 years. Two years after the end of maintenance therapy he presented with isolated bone marrow relapse. In the absence of suitable donor, he was treated with aggressive chemotherapy without HSCT. His treatment was complicated with PRES and pulmonary aspergillosis. Still on maintenance therapy, at the age of 11 years, isolated bone marrow relapse occurred. Remission was achieved with two courses of Blinatumumab, with SIRS during infusion managed with dexamethasone, inotrope support and single dose of Tocilizumab. Therapy with Blinatumumab was complicated with paranasal sinus and pulmonary aspergillosis and managed with systemic antifungal therapy and surgery. He proceeded to HSCT with HLA-A mismatched donor, conditioning regimen included TBI and Cyclophosphamide, GvHD prophylaxis consisted of Cyclosporine and Methotrexate. At the time of engraftment (day 22) patient developed fulminant acute lung injury, requiring prolonged mechanical ventilation for 8 weeks. Open biopsy excluded aGvHD and acute pulmonary hemorrhage, repeated bronchoalveolar lavage specimens remained negative for bacteria, viruses, PCP and fungi. Nonspecific toxic lung injury was final diagnosis. Simultaneously generalized erythroderma with bullous formation signaled grade IV aGvHD evolution with confirmatory skin biopsy. High dose systemic corticosteroids were introduced (Methylprednisolon 2 mg/kg iv), but without major skin improvement and gradual improvement of lung function. MSC infusions were started on day +55 to allow rapid tapering of corticosteroids that were not effective for skin aGvHD. Intensive schedule, as proposed by Osiris sponsored phase 3 trial for steroid refractory aGvHD, with infusion of 2 × 10^6^ MSC/kg twice weekly 4 weeks and then once weekly for another 4 weeks, has been introduced. This time MSC from unrelated donor were available in 1 week after clinical indication was recognized by treating transplant physician.

After 3rd infusion of MSC remarkable improvement of skin was noticed, total 10 infusions of MSC were given to complete resolution of aGvHD. Extracorporal photopheresis was started on day +89 to maintain remission, Cyclosporine was tapered (day +135). Patient remained in complete remission of ALL and GVHD without signs of active fungal or viral infections.

### Methods

MSC were derived from haploidentical (first patient) or unrelated (second and third patient) donors. Before collection, donors were screened for the absence of infections and assessed for suitability by expert clinician.

The whole procedure for preparation of clinical grade MSC was performed under good manufacturing practice conditions, needed for cell cultivation as approved by Agency for Medicinal Products and Medicinal Devices of the Republic of Slovenia. The overall process was approved by national Ethics committee and each patient was presented and approved to receive MSC therapy by the institutional review board and national competent regulatory authorities (Agency for Medicinal Products and Medicinal Devices of the Republic of Slovenia).

Allogeneic human AB serum was prepared by Blood transfusion center of Slovenia according to all standards in transfusion medicine and compliant to Slovenian national laws. Sera from three healthy donors were pooled and pool of sera was used for cell cultivation.

Cells were isolated and cultivated as previously described (Le Blanc et al., 2004; Kuçi et al., 2016). Briefly, cells were isolated from bone marrow with gradient centrifugation (Ficoll-Paque™ PREMIUM, GE Healthcare), washed with DPBS (Gibco) and seeded to Multi flask 3 or 5-layer (Falcon)at a density of 200,000 TNC per cm^2^ of tissue culture flasks. Cells were grown in D-MEM/F-12 (Gibco) medium supplemented with 10% alogenic human serum, bFGF (PeproTech). Gentamicyn (Gibco) and Amphotericin B (Gibco) were used only in primary culture.

Cells were cultivated at 37°C in a humidified atmosphere containing 5% CO_2_ until 80% confluent, when they were harvested using TrypLE™ Select CTS™ (Gibco), counted with Trypan Blue (Fluka) and reseeded in fresh medium at 5,000–10,000 cells/cm^2^. Final product was prepared by resuspension of cells in physiological solution supplemented by human serum albumin in a concentration of 1 × 10^6^ cells per milliliter of solution. Final product was stored at room temperature and used within 4 h after preparation.

When not cultivated, cells were cryopreserved in gaseous phase of liquid nitrogen. Cell pellet was re-suspended in allogeneic human serum supplemented with 10% DMSO and transferred to vessels for controlled cryopreservation (−1°C per minute) until −80°C was achieved and subsequently transferred to liquid nitrogen (<−130°C).

Cell products were monitored by internal and external quality control.

Criteria for release of MSC for clinical use included normal spindle shaped morphology and absence of cell clumps (internal inspection), absence of microorganism contamination including mycoplasma (internal PCR and external cultivation testing according to European Pharmacopeia), endotoxin levels in the final product ≤ 2.5 EU/ml (external testing), viability >95% (internal and external counting), and immune phenotyping (external testing of presence of CD73, CD90 and CD105 and absence of CD34 and CD45 surface antigens).

Cells were given fresh as intravenous infusions through standard hematological filter for removing cell clumps in 15–30 min bolus and patients were closely inspected for any adverse events during, 24 h after infusions and routinelly inspected daily by clinicians.

## Results

**Figure 1:**
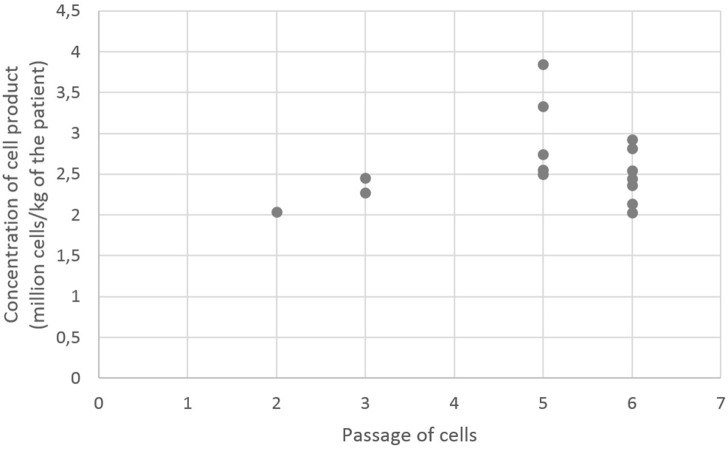
Numbers of cells given to patients (million cells per kilogram of patients weight) and passagesof cells.

A total of 15 infusions of MSC were given treating three patients with steroid refractory aGvHD (lack of steroid responsiveness for at least 5 days) after approval of the regulatory authorities in single pediatric transplant center. Numbers of cells given to patients (million cells per kilogram of patient's weight) and passages of cells are shown in Figure 1. Median dose of MSC was 2.5 × 10^6^ MSC per kg BW (range 2.0–3.8 × 10^6^ MSC per kg BW). One patient received two, one three, and one ten infusions of MSCs.

Two patients had complete resolution (CR) of aGvHD after 2nd and 3rd MSC infusion, one patient had partial response (PR). Median response time to MSC infusion was 7 days.

Initial MSC application was not as intensive as we would like due to production difficulties and lack of established clinical guidelines. First patient received MSC after several lines of immunosuppression in wide intervals. Third patient was treated with MSC infusion shortly after aGvHD was recognized as steroid refractory. Infusion regimen was planned ahead in concordance with recently published trials.

General performance status of the first patient was poor at the time of MSC infusion, because of long lasting GvHD and toxicity of previous therapies. Previously acquired infectious complications were cause of death despite partial response to MSCs. aGvHD presented late in second patient due to intensive immunosuppression with high dose systemic corticosteroids and Etanercept for IPS early after HSCT. Pulmonary fungal infection was present prior to first aGvHD manifestation, but progressed to CNS during further immunosuppression with first line therapy for aGvHD. Patient had a complete response to MSC infusion, progressive CNS fungal infection being immediate cause of death.

Both patient developed invasive fungal infection and viral reactivation before MSC infusion. Therefore, MSC administration was not considered as contributing factor in those infections.

In third patient fungal infection was managed more aggressive with combination of surgery and systemic therapy, which together with complete response to MSCs resulted in long term survival (30 months) without signs of acute or chronic GvHD. Patient achieved prompt remission of leukemia with recovering neutrophils on Blinatumumab, enabling recovery from invasive fungal infection. Acute GvHD was limited to skin.

All three patients remained in remission of their primary disease.

We recorded one acute and no delayed adverse effects of MSC infusions. Second patient required transient oxygen supplementation after first MSC infusion, grade 1 adverse effect considered not to be related to treatment with MSC. Patient had uncontrolled pulmonary aspergillosis at that time.

Patient's characteristics, treatment prior to MSC, detailed data regarding MSC infusion and response are summarized in Table 1.

**Table 1:** GvHD grade, organ involvement, therapies used prior to MSC application, dosage and response to MSCs.

**Patient no**.	**1**	**2**	**3**
GvHD grade (organ involvement)	III (skin GIT)	III (skin GIT)	IV (skin)
Onset of GvHD post HCST/duration of GvHD prior to MSC	65/173	238/280	22/55
Prior therapy for GvHD	Methylprednisolone (2 mg/kg) MMF Tacrolimus Alemtuzumab Infliximab ECP Sirolimus MTX Imatinib	Methylprednisolone (2 mg/kg)	Methylprednisolone (2 mg/kg)
Response	PR	CR	CR
No of MSC infusions/cummulative dose of MSC (average MSC cells/kg/infusion)	3/7 × 10^6^ (2.3 × 10^6^)	2/4, 7 × 10^6^ (2.4 × 10^6^)	10/27, 3 × 10^6^ (2.7 × 10^6^)
Patient outcome	Dead with signs of cGvHD	Dead in CR	Alive in CR (8 months post HSCT)

Final cell products all exhibited absence of microorganisms, bacterial endotoxins and expressed normal spindle shaped morphology and normal cell growth.

The viability of MSC in final cell products was >95% in all but one cell product (where it was 93.9%) and was thus regarded as suitable.

The phenotype of the cell products revealed majority of cells expressed common MSC antigens, namely CD73 (>95% in 13 products and >68% in the rest), CD 90 (>95% in 12 products and >70% in the rest) and CD 105 (>95% in 13 products and >65% in the rest). Similarly, majority of cell products had very low percentage of CD 34 and CD 45 cells (<1.3 and <0.3% respectively). First cell product for first patient had lowest percentage of MSC antigens, since clinical status of patient was dictating cell production.

## Discussion

We provide detailed description of MSC generation and application in small country with single pediatric transplant center. Small number of patients allows no conclusions about efficacy, acute or late effects of MSC infusions for treatment of steroid refractory aGvHD. The effect MSC had in our small series (2 CR and 1 PR) is concordant with published results from some larger studies in the past and present report from group of P Bader, showing MSC as a as feasible and effective therapy for steroid refractory aGvHD (Baron and Storb, 2012; Herrmann et al., 2012; Lalu et al., 2012; Chen et al., 2015; Bader et al., 2018).

Due to low number of patients, in our case series no patient had signs of aGvHD, affecting the liver. It is well established, that skin aGvHD responds somewhat better to MSC administration than aGvHD of gut and liver (Kurtzberg et al., 2014; Chen et al., 2015). The absence of liver aGvHD in our case series should thus be taken into account. All three responses to MSC trearment in our case series accounted for skin and gastrointestinal aGvHD.

Despite high response rate in our small case series, it is crucial to constantly improve the cell products in order to further improve response rate for the patients. Fortunately, there are some recent advances in preclinical field that have potential to affect the field. One of them could be recent theory, that MSCs in apoptosis could be more effective than viable MSCs (Galleu et al., 2017). Other possibilities include different cell sources and dose dependent studies.

We managed to manufacture safe, potent and quality cell based therapy in 3 months. Our clinical experience grew with number of patients treated. Early treatment of steroid refractory aGvHD with MSC could prevent occurrence of life threating bacterial, viral and fungal infections.

Recently published study confirmed MSC as an excellent salvage therapy for both steroid and treatment refractory aGvHD. Its large cohort of patients with high grade aGvHD and use of standardized product is a great advantage. Results showed superior 6-months OS rate in range of 61–83% compared to patients not treated with MSC; survival appeared similar to patients without severe aGvHD. The study showed no difference in efficacy between children and adults and in steroid or treatment refractory patients (Bader et al., 2018).

In conclusion, stored pool of MSC should be available for patient when receiving HSCT, since prompt therapy with MSC has potential to adapt to patient's environment and orchestrate immune system back in order in case of severe steroid refractory aGvHD. Only detailed description of patient's features and production protocol will enable comparison and evaluation of cell-based therapies.

## Ethics statement

This study was carried out in accordance with the recommendations of Slovenian national ethical committee. The protocol was approved by the Slovenian national ethical committee. All subjects gave written informed consent in accordance with the Declaration of Helsinki.

## Author contributions

MĆ had primary responsibility for protocol development, patient screening, enrollment, outcome assessment, final data analysis, and manuscript writing. LG had primary responsibility for cell cultivation protocol development, final data analysis and manuscript writing. MK participated in cell cultivation protocol development and contributed to writing of the manuscript. SA participated in patient screening, enrollment, outcome assessment, and manuscript writing. JJ supervised the design and execution of the study, participated in enrollment and contributed to manuscript writing.

### Conflict of interest statement

The authors declare that the research was conducted in the absence of any commercial or financial relationships that could be construed as a potential conflict of interest.
